# Preparation of Device-Level ZnO-Covered Silver Nanowires Films and Their Applications as Sub-Electrode for Polymer Solar Cells

**DOI:** 10.3389/fchem.2021.683728

**Published:** 2021-09-24

**Authors:** Xin Zhao, Meng Li, Linping Jiang, Hua Tang, Youwei Guan

**Affiliations:** ^1^ Graduate Department, Civil Aviation Flight University of China, Guanghan, China; ^2^ School of Materials Science and Engineering, Chongqing University of Arts and Sciences, Chongqing, China

**Keywords:** silver nanowires films, AgNWs, transparent conductive films, polymer solar cells, organic photoelectronic devices

## Abstract

Silver nanowire films are good candidates to be used as transparent conductive films that could be widely utilized in organic photoelectronic devices such as polymer solar cells. However, their application is usually limited, as they are mainly used as top electrode materials; otherwise, they would be prone to complex transferring processes. In this study, we successfully prepared device-level ZnO-covered silver nanowire (AgNWs/ZnO) films. ZnO was prepared by a spray pyrolysis method using zinc-ammonia solution at a relatively low temperature (95°C). The films showed good adhesive properties to the glass substrate, considering it withstood the process of applying polyimide tapes on the surface and tearing them off more than 100 times. It also exhibited good conductivity (∼24 Ω/sq) with high transmittance in the visible range (>80%). After a simple polish and patterning, AgNWs/ZnO showed a good performance as a sub-electrode for polymer solar cells. The PM6:Y6 devices achieved a high power conversion efficiency of 8.37% with an open-circuit voltage of 0.81 V, a short-circuit current density of 18.18 mA/cm^2^, and a yield of 81.25%. This indicates that the technology has a good prospect of large-scale fabrication of organic photoelectronic devices.

## Introduction

Polymer solar cells (PSCs) have gained much interest due to their lightweight, flexibility, and the possibility to fabricate them by solution-processing methods at a low cost (Zheng et al., 2018; Song and Bo, 2019; Cui et al., 2020). Recently, single-junction polymer solar cells, based on non-fullerene acceptors, have achieved outstandingly high efficiency (>17%) (Meng et al., 2018; Cui et al., 2020). It may be the right time to consider optimizing relevant industrial fabrication methods. Strenuous efforts have been made to achieve this goal. A lot of large-scale PSCs have been fabricated using doctor-blading, brush-painting, screen printing, and spray coating, and the outcomes are quite promising. For example, Fan et al. obtained a PM6:IDIC-based flexible PSC with a large device area of 1.25 cm^2^, which exhibited a high power conversion efficiency (PCE) of 6.54% (Fan et al., 2018). Cheng et al. (2018) also managed to develop a large-scale PBDB-T-2Cl:IT4F module with a satisfying PCE of 6.68%. Despite these great improvements, we are still far from the mature commercial production of PSCs. One of the most crucial problems is that the costs of the materials and the preparation process are still too high to meet the requirements of the photovoltaic market (Zimmermann et al., 2007; Cui et al., 2019). Among the materials used in PSCs, including organic semiconductors, buffer materials, and electrode materials, transparent electrode materials constitute a considerable portion of the cost. The application of indium tin oxide (ITO), which is a widely used transparent conductive film (TCF), is limited by the rarity of indium (Cheng et al., 2014; Masai et al., 2015). Although other TCFs, such as SnO_2_:F (FTO), ZnO:Al (AZO), and graphene films (Baek et al., 2010; Wei et al., 2016; Aimon et al., 2019), consist of much cheaper elements, their dependence on vacuum processing also represents an additional cost.

In the past few years, composite films based on silver nanowire (AgNWs) networks have been recognized as a promising substitute for ITO. This is because of their high electronic conductivity, high optical transparency, and low-cost fabrication (Chen et al., 2018; Wang et al., 2019). However, the roughness of directly coated AgNWs films is relatively high considering the thickness of an active layer (∼200 nm). In many works, AgNWs films were used as the top electrode, and the ITO glass substrate could not be substituted (Khatri et al., 2014; Zhang et al., 2016). To reduce the roughness to an acceptable level, the peeling-off method is needed. In a typical process, an AgNWs film is first coated on a smooth plate (e.g., glass). Then, a glue solution containing an epoxy or polyurethane monomer and a curing agent is subsequently coated on the AgNWs film. After a thermal or UV curing, the AgNWs-containing composite film could be torn off from the smooth plate. AgNWs-containing TCFs have been widely used in flexible and stretchable OLED and OPV devices by many groups (Li et al., 2011; Liang et al., 2015; Bai et al., 2018). An obvious disadvantage of the films prepared by this method is the difficult post-patterning, which is not applicable on a large scale and for the fabrication of devices with complex structure. Furthermore, it is complex and time consuming.

It is well known that ZnO films are usually used as a cathode buffer layer (CBL) in both polymer solar cells and OLEDs for their suitable energy levels (Sun et al., 2011; Liang et al., 2012; Noh et al., 2013). AgNWs/ZnO composites, as transparent electrodes, have already been employed in photoelectric devices by a few groups. Han et al. (2018) developed a perovskite solar cell that reached a high PCE of 13.27% using an AgNWs/ZnO composite as the top electrode. The AgNWs and ZnO were prepared by spray coating using dispersions containing AgNWs and ZnO nanoparticles, respectively. The poor adhesion of ZnO nanoparticles and AgNWs to the cathode buffer layer could be an important factor that restricted its charge transport and performance. To improve it, Kim et al. (2013) designed a ZnO/AgNWs/ZnO structure as the top electrode for Cu(In,Ga)(S,Se)_2_ (CIGSSe) solar cells, in which ZnO was prepared by direct current magnetron sputtering. The adhesion properties showed an increase of 20% compared to that of ITO electrodes. However, this routine seems inefficient and uneconomic, because only a thick AZO layer deposited by sputtering could work well. AgNWs/ZnO structures could also be used as sub-electrodes for photoelectric devices by depositing a ZnO layer on AgNWs films prepared by the peeling-off method (Li et al., 2011; Liang et al., 2015; Bai et al., 2018). However, because of the complex process, the application is still very limited.

Until now, the realization of a device-level AgNWs/ZnO film using a simple process has been our main goal. Thus, we developed a solution-based method to prepare AgNWs/ZnO films as simply as possible that can adhere well to a substrate with a smooth surface. We successfully achieved this by spray coating ZnO on AgNWs films using a zinc-ammonia solution at 95°C that we discussed previously (Cheng et al., 2016; Zhao and Cheng, 2016). The films could easily be patterned; they had a smooth surface after polishing, which could be used in both rigid and flexible devices. This technique is quite simple and is much more efficient than the aforementioned peeling-off method. Using transfer-free silver AgNWs/ZnO films, we achieved a high PCE of 8.7% for the well-known PM6:Y6 solar cells, indicating that they have a good prospect of large-scale solar cell fabrication.

## Results and Discussion

**FIGURE 1:**
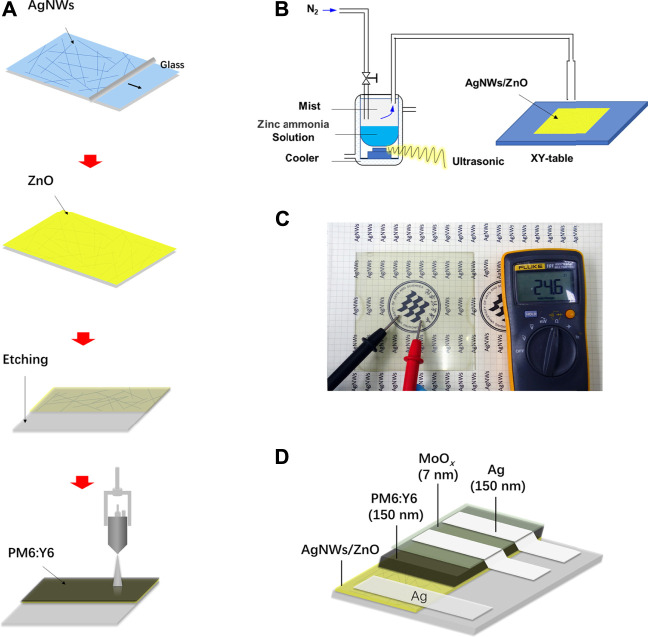
Preparation of AgNWs/ZnO and PM6:Y6 solar cells. **(A)** Preparation of AgNWs/ZnO and PM6:Y6 blend films, **(B)** a schematic image of the spray pyrolysis apparatus, **(C)** a photograph of the prepared AgNWs/ZnO films deposited on glass, and **(D)** structure of the polymer solar cells situated on a AgNWs/ZnO film.

A schematic image of the preparation of AgNWs/ZnO films and PM6:Y6 active layers is shown in Figure 1A. ZnO was deposited on AgNWs using a spray pyrolysis apparatus (Figure 1B). The ultrasonic generator with a frequency of 1.44 Hz could make microdroplets with a diameter of about 10–20 μm from the zinc-ammonia solution. Then, the microdroplets were condensed and pyrolyzed onto the AgNWs layer at 95°C. The prepared AgNWs/ZnO films were rather transparent with high electrical conductivity (Figure 1C). The PSCs based on AgNWs/ZnO electrodes were fabricated on glass, giving the final structure of the glass/AgNWs/ZnO/PM6:Y6/MoO_3_/Ag system, as shown in Figure 1D.

**FIGURE 2:**
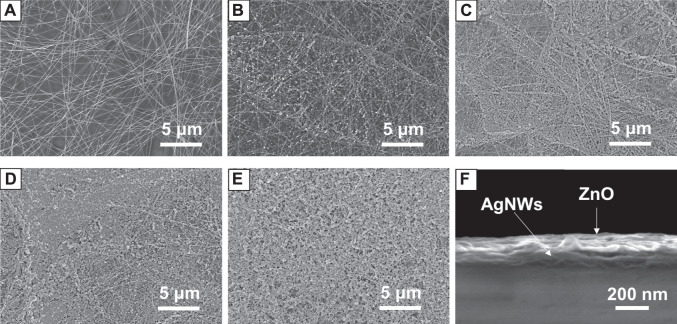
Morphology of AgNWs/ZnO films. SEM micrographs of **(A)** AgNWs films and AgNWs/ZnO films with ZnO thicknesses of **(B)** 30 nm, **(C)** 60 nm, **(D)** 90 nm, and **(E)** 120 nm; **(F)** cross-section view of a AgNWs/ZnO film with a 90 nm-thick ZnO.

**FIGURE 3:**
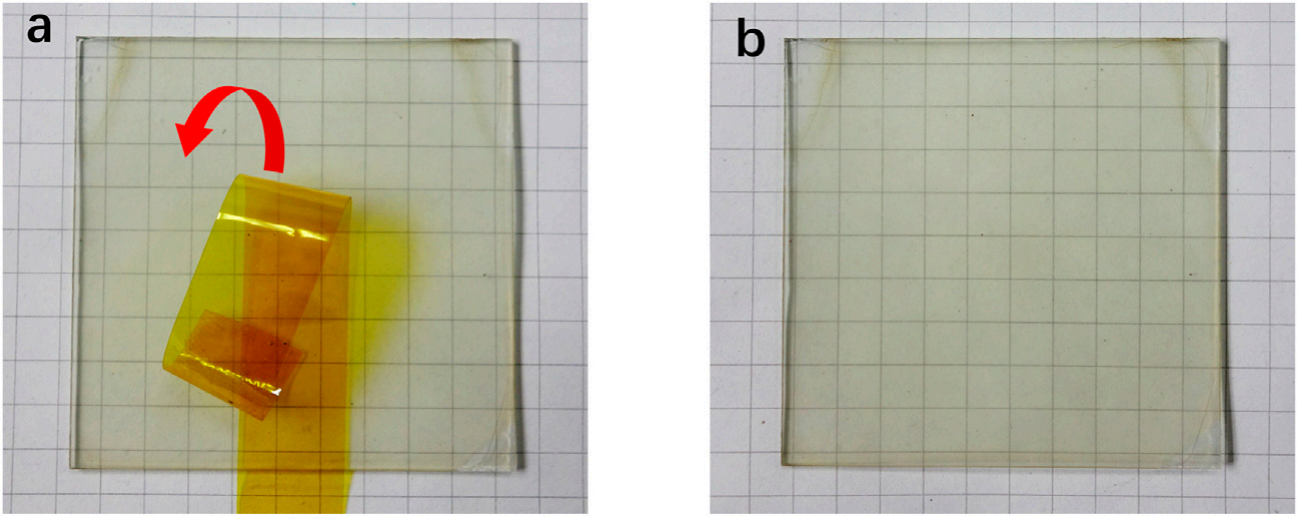
Adhesion tests of AgNWs/ZnO on a glass substrate. AgNWs/ZnO film **(A)** before and **(B)** after sticking and tearing the tape off 100 times.

To investigate the morphology of the AgNWs/ZnO samples, ZnO films of different thicknesses were measured by SEM. Figures 2A–E show SEM micrographs of the AgNWs by themselves or covered with 30–120 nm-thick ZnO. The thickness of ZnO was determined as it was prepared on glass under the same conditions, and the deposition rate of ZnO was 30 nm/spray cycle. In Figure 2A, the AgNWs deposited on glass with an average length of 100 μm and a diameter of 20 nm could be clearly distinguished. After 30 nm of ZnO was deposited, ZnO nanoparticles were found to evenly adhere to the AgNWs. As the number of spray cycles was increasing, the AgNWs were gradually covered by the ZnO nanoparticles. After 90 nm of ZnO was deposited, only traces of AgNWs could be found in the films. When the number of spray cycles was increased above four, almost all of the AgNWs were covered deeply in the ZnO, as shown in Figure 2E. From the cross-section view of the AgNWs/ZnO (Figure 2F), spray-pyrolyzed ZnO and AgNWs can be seen strongly attached to each other and adhered to the glass substrate. We used polyimide tapes to test the adhesive properties of the AgNWs/ZnO to the glass substrate. After the tapes were applied to the surface and torn off more than 100 times, the AgNWs/ZnO film was still firmly adhered to it and no trace of damage could be observed, as shown in Figures 3A,B.

**FIGURE 4:**
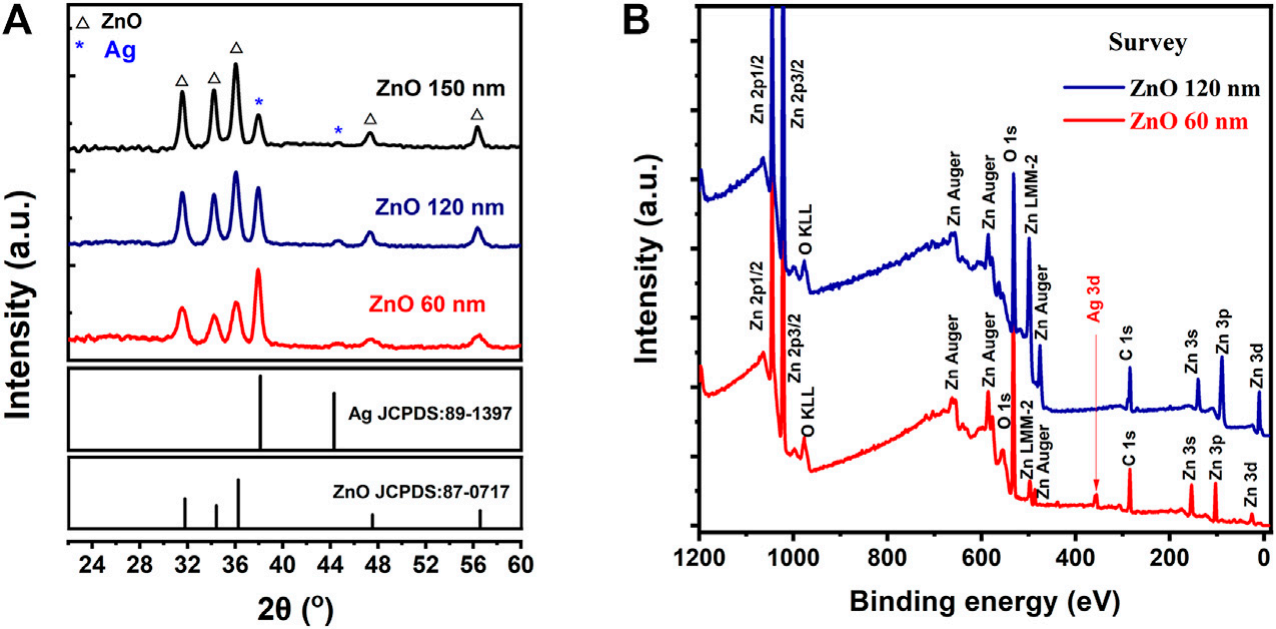
Composition and structural analysis of AgNWs/ZnO films. **(A)** XRD patterns and **(B)** XPS survey spectra of AgNWs/ZnO films with 60 nm- and 120 nm-thick ZnO.

The composition of AgNWs/ZnO films was investigated by XRD. Figure 4A shows the XRD patterns of AgNWs/ZnO films with 60 nm- and 120 nm-thick ZnO. Both samples had mixed phases. The ZnO and Ag diffractions peaks were easily distinguishable, which were attributed to the NO.811397 and NO.870717 JCPDS cards, respectively. This indicated that the zinc-ammonia solution completely decomposed to ZnO. No other diffraction peaks were found in both samples, demonstrating that the AgNWs were stable and unoxidized during the decomposition and pyrolysis of zinc-ammonia at 95°C. The stability of AgNWs contributed to the good electric and optical properties of AgNWs/ZnO films, which is beneficial to their application as a transparent electrodes material. XPS spectra of the AgNWs/ZnO films of different thicknesses were recorded to further access the surface condition (Figure 4B). For the films with 60-nm-thick ZnO, only a few weak Ag XPS peaks could be found corresponding to AgNWs at a binding energy of 355 eV. This indicated that most of the AgNWs were wrapped in ZnO. When the thickness of ZnO was increased to 120 nm, the Ag peaks completely disappeared, demonstrating that the surface state was dominated by ZnO that was deposited by spray pyrolysis.

**FIGURE 5:**
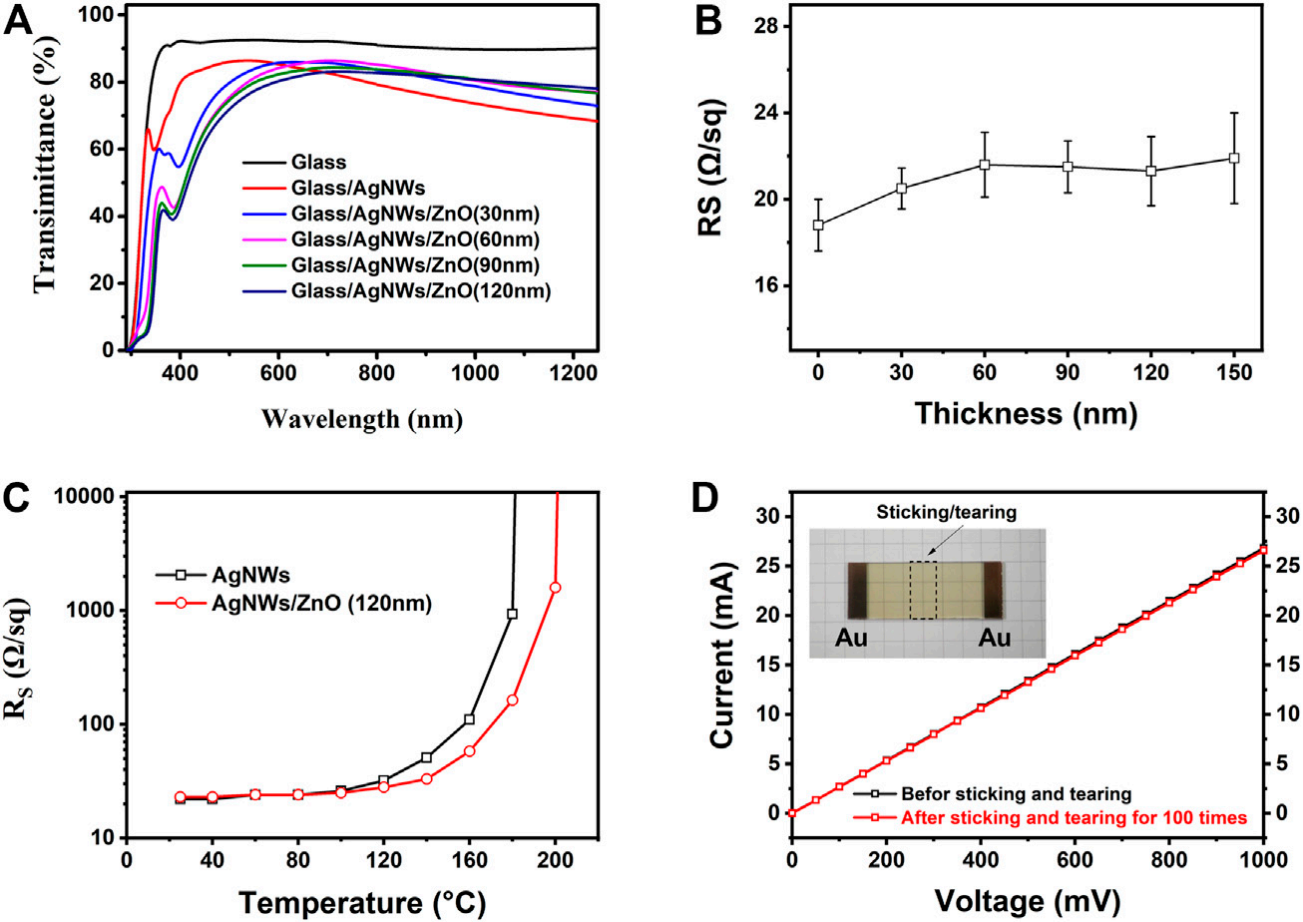
Optical and electrical analysis of AgNWs/ZnO films. **(A)** UV-Vis-NIR spectra and **(B)** sheet resistance of the samples with different ZnO thicknesses, **(C)** sheet resistance of two samples treated at different temperatures, and **(D)** current–voltage curve of a module.

The optical and electrical properties of the AgNWs/ZnO electrodes were measured by UV–Vis–NIR spectrophotometry and four-point probe sheet resistance measurements. Figure 5A shows the UV-Vis-NIR spectra of the AgNWs/ZnO films of different ZnO thicknesses. The glass substrate showed a high transmittance of about 90% in the visible- and near-infrared ranges. The AgNWs film without ZnO exhibited high transmittance, greater than 82%, between 400 and 600 nm, with a drop in transmittance at longer wavelengths due to absorption by surface plasmons (Li et al., 2009; Li et al., 2015). All the AgNWs films with ZnO exhibited a drop in transmittance at shorter wavelengths (400–550 nm). However, since ZnO modified the surface state of the AgNWs, the surface plasmon effect was obviously reduced. Thus, this manifested in higher transmittances at higher wavelengths (600–1,200 nm) compared to that of the samples without ZnO. The high transmittance at higher wavelengths facilitates the enhancement of the device’s performance for organic photovoltaic devices. This is because organic solar cells usually have a relatively high photoresponse in the visible range. Figure 5B shows the sheet resistance of the AgNWs/ZnO films of different ZnO thicknesses. The AgNWs films without ZnO exhibited a low average sheet resistance (R_S_) of about 18.6 Ω/sq, while all the AgNWs films with ZnO showed a slight increase. The R_S_ of the AgNWs/ZnO films with 30 nm-thick ZnO was only 20.5 Ω/sq. Even when the thickness was increased to 150 nm, the R_S_ of the AgNWs/ZnO films was still lower than 24 Ω/sq. The thermal stability of the AgNWs film could be enhanced by covering a ZnO film. As shown in Figure 5C, the R_S_ of the AgNWs film without ZnO increased rapidly when the temperature was higher than 120°C. However, the AgNWs film with a 120 nm-thick ZnO still showed a low R_S_ of 33 Ω/sq when the temperature reached 140°C. This enhancement in the stability could also prolong the stability of the AgNW film-based photoelectric device. The adhesive properties of the AgNWs/ZnO were also investigated by electrical tests. An 80 × 15 mm^2^ module was prepared using a 120-nm thick ZnO film. Both ends were deposited on an approximately 100 nm-thick Au electrode by direct current sputtering. The resistance of the module before and after applying and tearing off polyimide tapes 100 times was measured by voltammetry, and the results are shown in Figure 5D. Almost completely overlapped current–voltage curves were observed before and after this test. Only a slight growth of resistance was detected from 37.2 to 37.6 Ω, showing its great adhesive properties to the substrate.

**FIGURE 6:**
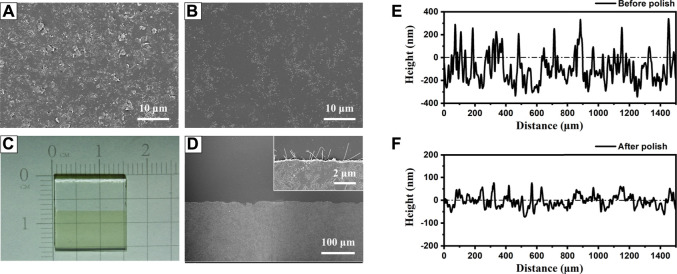
Polishing and etching treatments of AgNWs/ZnO films. SEM micrograph of AgNWs/ZnO films **(A)** before and **(B)** after polishing; **(C)** photograph of patterned AgNWs/ZnO films and **(D)** top view of the etched edge. Surface topography of AgNWs/ZnO films **(E)** before and **(F)** after polishing.

**FIGURE 7:**
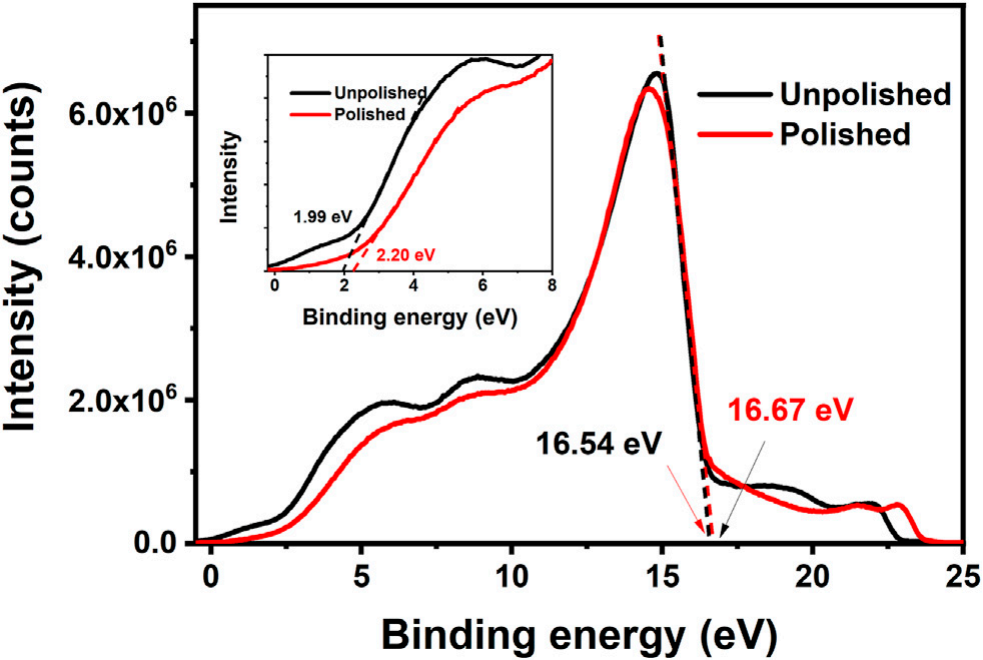
UPS spectra of samples before and after polishing.

A preliminary polishing process is necessary for the AgNWs/ZnO films to be used as a substrate electrode because the thickness of the active layer was always lower than 200 nm and a smooth surface is required to avoid short-circuit (Cheng et al., 2018; Cheng et al., 2020). Figures 6A,B show SEM micrographs of an AgNWs/ZnO film. The unpolished film showed a rough surface, and big ZnO aggregates were found all over the film. After a simple polish for several minutes, the surface became much smoother. The surface undulation was measured with a profilometer, and the results are shown in Figures 6E,F. Unpolished samples exhibited large uneven regions on the surface (±250 nm), which became much flatter (±80 nm) after the polishing. The polishing had no obvious effect on the surface energy level of the films. As shown in Figure 7, the differences in cut-off edge of ultraviolet photoelectron spectra (UPS) between samples before and after polishing are very small. The work function (WF) value that calculated from UPS is 4.67 and 4.54 eV for unpolished and polished sample, respectively. Before application in an organic photovoltaic device, an AgNWs/ZnO substrate electrode should be patterned. Figure 6C shows a photograph of a patterned AgNWs/ZnO film that was etched using dilute hydrochloric acid. Based on the SEM micrograph (Figure 6D), the edge was very distinct. Some AgNWs protrusions were found in the magnified SEM image, indicating that they were wrapped in the ZnO film.

**FIGURE 8:**
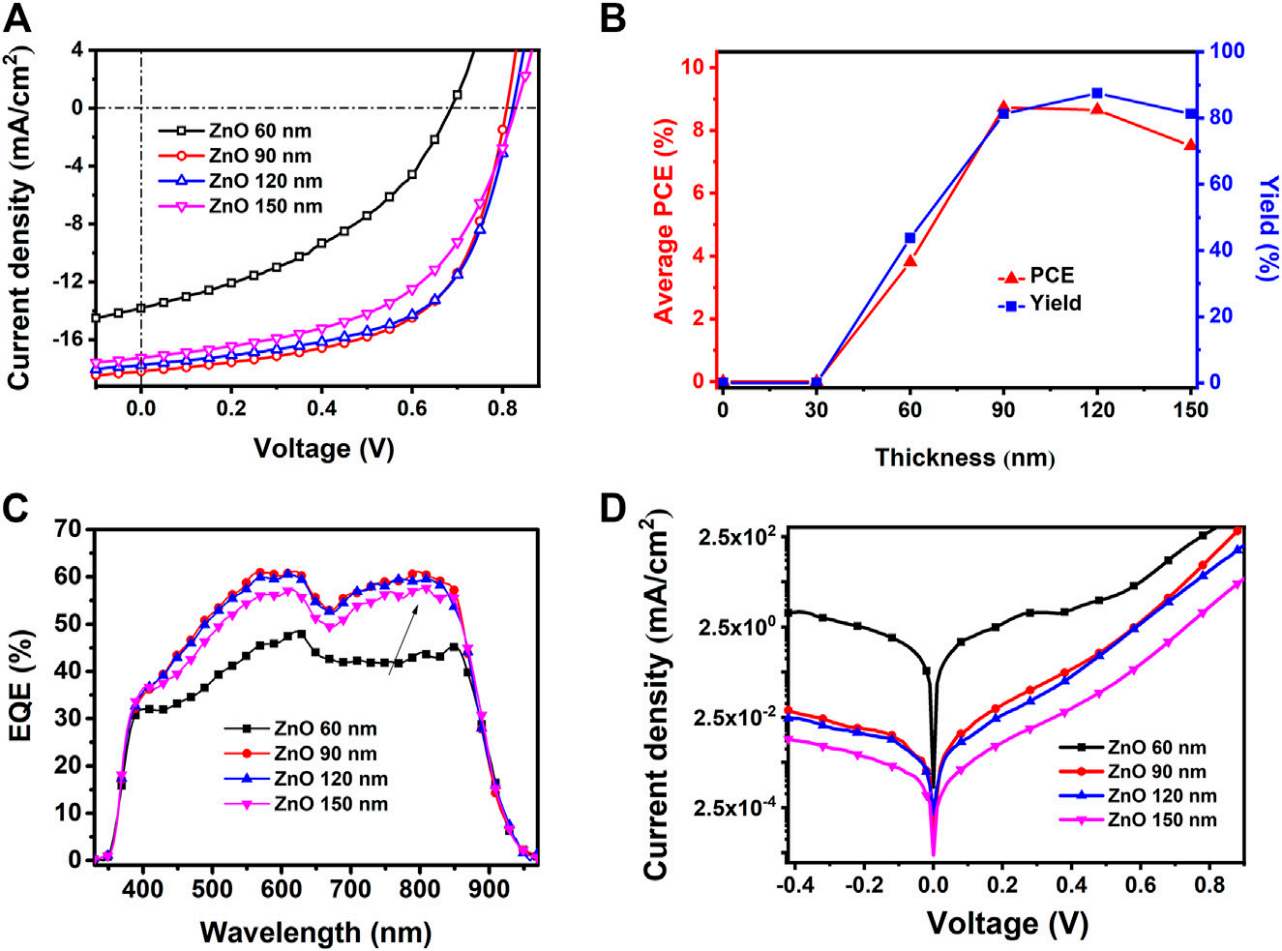
Performance of spray-coated devices based on AgNWs/ZnO films of different ZnO thicknesses. **(A)** J–V characteristics under irradiation, **(B)** yields, **(C)** EQE curves, and **(D)** J–V characteristics of the samples in the dark.

**FIGURE 9:**
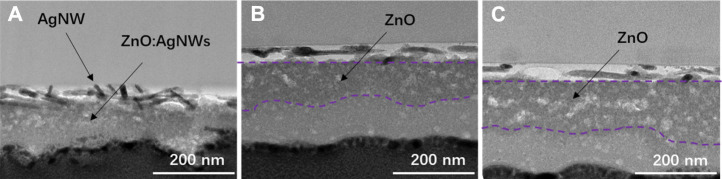
Cross-section investigation of the AgNWs/ZnO films. TEM images of films with **(A)** 90 nm, **(B)** 120 nm, and **(C)** 150-nm-thick ZnO.

To evaluate the practical effect of the AgNWs/ZnO films, they were used to prepare polymer solar cells, whose structure is shown in Figure 1D. The sheet resistance and transmittance of the AgNWs/ZnO films before polishing (BP) and after polishing (AP) were measured and the results are listed in Table 1. The optical and electrical properties of all polished samples showed a slight improvement. The total thickness (δ_A_) of the AgNWs/ZnO film containing a 120 nm-thick ZnO was 166 nm, which was decreased to 133 nm after polishing. The average transmittance (T_A_) and average Rs were increased to 82.1% and 21.8 Ω/sq, respectively, which are slightly better than those of the unpolished sample. To evaluate their yield, four specimens were prepared under the same conditions, and each specimen had four cells with an active area of 0.06 cm^2^. The effect of ZnO thickness (30–150 nm) on the device was also investigated. We found that all the devices without ZnO or with ZnO of less than 30 nm thicknesses were almost completely ineffective, and even the best PCE was lower than 0.1%. However, above 60 nm thicknesses, the devices showed much improved performances. The J–V characteristics under AM 1.5G irradiation for typical devices of 60, 90, and 120 nm-thick ZnOs are shown in Figure 8A, the average PCEs and yields of devices with different thick ZnOs are along shown in Figure 8B, and the detailed performances of the devices are summarized in Table 2. Device 1, with a thickness of 60 nm, showed an open-circuit voltage (V_OC_) of 0.69 V, a circuit current density (J_SC_) of 13.83 mA/cm^2^, and a fill factor (FF) of 40.35%, resulting in a low PCE of 3.82%. Moreover, the devices prepared with the same procedure had significant differences. If a “qualified sample” is defined as a device that has a PCE higher than 80% of the best PCE, the yield of the device with 60-nm-thick ZnO was only 43.75%. When the thicknesses of ZnO were 90 and 120 nm, the number of short-circuited devices was significantly reduced, and their yield was improved to about 81.25 and 87.50%, respectively. The typical performance of the devices with 90- and 120-nm-thick ZnOs was quite similar. For example, Device 3 had the highest PCE of 8.37% with a V_OC_ of 0.81V, a J_SC_ of 18.18 mA/cm^2^, and a high FF of 59.37%. However, when the ZnO thickness was above 150 nm, the performance was clearly lower. The PCE of Device 4 was only about 7.51%, which is much lower than those of Device 2 and Device 3. Figures 9A–C are the transmission electron microscope (TEM) images of the AgNWs/ZnO films with 60-, 120-, and 150-nm-thick ZnO, respectively. As we can see, many outcrops of AgNWs could be found in the film with 60 nm-thick ZnO, which are speculated to be some serious carrier recombination centers. The region that contained ZnO only in the film with 150-nm-thick ZnO is much thicker compared to the film with 120 nm-thick ZnO. As shown in Figure 2 and Figure 4A, ZnO exhibited polycrystalline characteristics with nanoscale grain size. The decrease in device performance was likely caused by the decreased resistance in the charge transfer circuit due to the larger number of ZnO grain boundaries in the thicker film.

**TABLE 1:** Thickness, transmittance, and sheet resistance of AgNWs/ZnO films in PM6:Y6 devices before polishing and after polishing.

Samples	ZnO thickness (nm) (BP)	δ_A_ (nm) (BP/AP)	T_A_ in visible (%) (BP/AP)	Transmittance at 550 nm (%) (BP/AP)	Average Rs (Ω/sq) (BP/AP)
Film 1	60	107/84	84.2/84.6	84.5/84.9	21.4/21.2
Film 2	90	139/110	82.2/83.6	84.7/85.3	21.7/21.6
Film 3	120	166/133	80.7/82.1	82.1/84.5	22.4/21.8
Film 4	150	198/162	78.6/81.0	80.8/82.2	23.3/22.7

**TABLE 2:** Performance of spray-coated typical devices based on AgNWs/ZnO films with different ZnO thicknesses.

Samples	ZnO thickness (nm)	V_OC_ (V)	J_SC_ (mA/cm^2^)	FF (%)	R_Se_ (Ω cm^2^)	R_Sh_ (Ω cm^2^)	PCE (%)
Device 1	60	0.69	13.83	40.35	15.46	121.84	3.82
Device 2	90	0.81	18.18	59.37	5.64	346.32	8.73
Device 3	120	0.82	17.75	59.25	6.71	435.72	8.65
Device 4	150	0.83	17.25	52.47	10.23	279.72	7.51

To confirm the effect of ZnO thickness on the performance of the devices, the external quantum efficiency (EQE) was measured and the spectra are shown in Figure 8C. All the EQE curves showed a similar shape compared to that reported in the publication of Yuan et al. (2019). The J_SC_ values calculated from the EQE spectra of the devices with 60, 90, 120, and 150-nm-thick ZnOs were 13.89, 18.07, 17.89, and 17.01 mA/cm^2^, respectively. These values were in good agreement with the J–V characteristics. The devices containing 90 and 120 nm-thick ZnO substrates showed the highest EQE (∼60%), while the device with 60 nm ZnO had a relatively low EQE of less than 45%. The device with 60-nm-thick ZnO had a much lower photoresponse in the 670–900 nm range. This suggested that considerable recombination occurred when the negative carriers were transported to the cathode because the photons with long wavelengths were largely absorbed near the Y6/ZnO interface (Yao et al., 2020). The J–V characteristics in the dark revealed that the device with 60-nm-thick ZnO had a much higher leakage current, as shown in Figure 8D. The leakage current could presumably be related to a facilitated charge carrier recombination caused by the poor coverage of ZnO on the AgNWs. This confirms that it is essential for the AgNWs/ZnO substrate to contain a thick enough ZnO layer when it is used as a substrate in photovoltaic devices.

**FIGURE 10:**
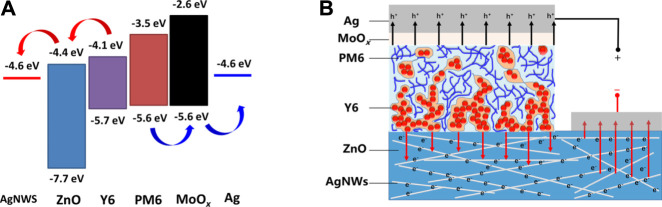
Carrier transport analysis of an AgNWs/ZnO-based polymer solar cell. **(A)** Energy level **(B)** and carrier transport path diagram of an AgNWs/ZnO-based PM6:Y6 polymer solar cell.

In this work, ZnO was used as a protective layer for the AgNWs; furthermore, it acted as an efficient electron transport layer. Thus, the devices based on the AgNWs/ZnO substrate electrode needed no more additional cathode buffer layers. The energy levels of the AgNWs/ZnO/PM6:Y6/MoO_3_/Ag heterojunction were depicted in Figure 10A. Because the conduction band minimum of ZnO is only slightly lower than the energy of the lowest unoccupied molecular orbital of Y6, electrons can be efficiently transported to the cathode without significant recombination (Ma et al., 2020). Generally, well-matched energy levels and a favorable contact at the interface enhance the charge transport properties and the overall performance of devices. It should be noted that the ZnO reported here could be much thicker than in previously reported works (Hu et al., 2016; Hu et al., 2018). Due to the AgNWs in the substrate that are practically immersed in the ZnO layer, the carrier transport properties are quite different from the conventional planar heterojunction. This structure is more beneficial for the transport of negative charge carriers from the acceptor material to the AgNWs network, which are then collected by the cathode. As shown in Figure 10B, the path for electron transport could be much shorter than that for planar structures when using the same ZnO.

## Experimental Section

### Chemicals

Zinc oxide (ZnO, 99.99%) nanopowder and ammonia (NH_3_•H_2_O, 25%∼28%) were purchased from Aladdin Inc. (Shanghai, China). Conjugated polymer PM6 and non-fullerene acceptor Y6 were purchased from Solarmer Material Inc. The AgNWs ink was purchased from Zhejiang Kechuang Advanced Materials Technology Co., Ltd. The average diameter of the AgNWs in the ink is 20 nm.

### AgNWs/ZnO Film Preparation

AgNWs underlayers were prepared by using a Meyer-rod coating equipment (Gardco, America). The AgNWs ink was diluted to 0.5 wt% before use. The roll speed of the Meyer-rod was set to 80 mm/s during the coating process. After that, the wet layer was naturally dried. During this process, the AgNWs layer had a moderate thickness with a transmittance of about 82–83% in the visible range and a sheet resistance of about 18–20 Ω/sq. ZnO was deposited on the AgNWs films by using a homemade ultrasonic spray pyrolysis apparatus. The deposition was carried out at a relatively low temperature (95°C) using a zinc-ammonia solution that was prepared by dissolving ZnO (0.3 wt%) in an ammonia–water mixture. Then, the ZnO was heat treated *in situ* at 120°C for 3 min. Additional details can be found in our previously reported works (Cheng et al., 2016; Zhao and Cheng, 2016). Then, the films were polished using nano-alumina polishing powder and were patterned by diluted hydrochloric acid. This was followed by washing with acetone and isopropanol in an ultrasonic bath, then by drying under N_2_ before using the films for device fabrication.

### Device Fabrication

In a typical process, a 150-nm-thick blend of PM6 and Y6 films in a weight ratio of 1:1.2 was spray coated on the AgNWs/ZnO film using an automatic spray apparatus with an ultrasonic nozzle (Z95S, Siansonic). Then, a 7-nm-thick MoO_3_ and a 150-nm-thick Ag film were successively deposited on the top of the PM6:Y6 film by thermal evaporation under a high vacuum (<10^−4^ Pa).

### Film Characterization

The morphology of AgNWs/ZnO was investigated by scanning electron microscopy (SEM, Zeiss Merlin, Germany). The composition and crystal structure of AgNWs/ZnO were characterized by X-ray diffraction (XRD, Bruker D8). Optical transmittance of AgNWs/ZnO was measured with a UV–Vis–IR spectrophotometer (Agilent Cary 5,000, America). Sheet resistance was tested using a four-probe system with a source meter (Keithley 2,400, America). The coverage of ZnO on AgNWs was analyzed by X-ray photoelectron spectroscopy (XPS, Thermo ESCALAB, America), and the thickness of the films was measured using a stylus profile meter (Alpha-Step D-100, America). The surface energy level of the films was analyzed by a UPS spectrometer (Thermo ESCALAB 250Xi, America). Cross-sections of AgNWs/ZnO films were observed by a transmission electron microscopy (JEM-F200 microscope, Japan). Before the test, TEM specimens were prepared by using a Zeiss cross beam 550L system with focused ion beam.

### Device Characterization

Current density–voltages (J–V) were measured under the dark and under illumination using a xenon lamp (94043A, Newport, America) with a power of 100 mW/cm^2^, which were recorded by a Keithley 2,400 source meter. The external quantum efficiency (EQE) of the devices was obtained using an integrated system (SOFN 7-SCSpecIII, China) with a lock-in amplifier under short-circuit conditions. The photovoltaic parameters (V_OC_, J_SC_, FF, and PCE) were calculated according to the J–V characteristics. The series resistance (R_Se_) and the shunt resistance (R_Sh_) of the devices were determined based on the reciprocals of the slopes of the J–V curves at their intersections with the abscissa and ordinate, respectively.

## Conclusion

In summary, we prepared device-level AgNWs/ZnO films by a spray pyrolysis method using zinc-ammonia solution at 95°C followed by heat treatment at 120°C. They showed good adhesive properties to the glass substrate, as it could withstand the process of applying polyimide tapes on the surface and tearing them off more than 100 times. The film exhibited good conductivity (∼24 Ω/sq) with high transmittance in the visible range (>80%). The films could be easily polished and easily patterned using dilute hydrochloric acid. As a transparent sub-electrode, it exhibited good performance when used in polymer solar cells after simple polishing and patterning. The PM6:Y6 devices based on AgNWs/ZnO with a 90-nm thick ZnO layer achieved a high PCE of 8.37%, a V_OC_ of 0.81 V, a J_SC_ of 18.18 mA/cm^2^, and a yield of 81.25%. This indicates that they have a good prospect of large-scale fabrication of organic photoelectronic devices.

## Data Availability

The raw data supporting the conclusions of this article will be made available by the authors, without undue reservation.
